# Heterologous prime-boost H1N1 vaccination exacerbates disease following challenge with a mismatched H1N2 influenza virus in the swine model

**DOI:** 10.3389/fimmu.2023.1253626

**Published:** 2023-10-20

**Authors:** Vasilis C. Pliasas, Peter J. Neasham, Maria C. Naskou, Rachel Neto, Philip G. Strate, J. Fletcher North, Stephen Pedroza, Strickland D. Chastain, Ian Padykula, S. Mark Tompkins, Constantinos S. Kyriakis

**Affiliations:** ^1^ Department of Pathobiology, College of Veterinary Medicine, Auburn University, Auburn, AL, United States; ^2^ Emory-University of Georgia (UGA) Center of Excellence for Influenza Research and Surveillance (CEIRS), Atlanta, GA, United States; ^3^ Center for Vaccines and Immunology, University of Georgia, Athens GA, United States

**Keywords:** influenza A virus, vaccine, swine, hemagglutinin, heterologous prime-boost

## Abstract

Influenza A viruses (IAVs) pose a significant threat to both human and animal health. Developing IAV vaccine strategies able to elicit broad heterologous protection against antigenically diverse IAV strains is pivotal in effectively controlling the disease. The goal of this study was to examine the immunogenicity and protective efficacy of diverse H1N1 influenza vaccine strategies including monovalent, bivalent, and heterologous prime-boost vaccination regimens, against a mismatched H1N2 swine influenza virus. Five groups were homologous prime-boost vaccinated with either an oil-adjuvanted whole-inactivated virus (WIV) monovalent A/swine/Georgia/27480/2019 (GA19) H1N2 vaccine, a WIV monovalent A/sw/Minnesota/A02636116/2021 (MN21) H1N1 vaccine, a WIV monovalent A/California/07/2009 (CA09) H1N1, a WIV bivalent vaccine composed of CA09 and MN21, or adjuvant only (mock-vaccinated group). A sixth group was prime-vaccinated with CA09 WIV and boosted with MN21 WIV (heterologous prime-boost group). Four weeks post-boost pigs were intranasally and intratracheally challenged with A/swine/Georgia/27480/2019, an H1N2 swine IAV field isolate. Vaccine-induced protection was evaluated based on five critical parameters: (i) hemagglutination inhibiting (HAI) antibody responses, (ii) clinical scores, (iii) virus titers in nasal swabs and respiratory tissue homogenates, (iv) BALf cytology, and (v) pulmonary pathology. While all vaccination regimens induced seroprotective titers against homologous viruses, heterologous prime-boost vaccination failed to enhance HAI responses against the homologous vaccine strains compared to monovalent vaccine regimens and did not expand the scope of cross-reactive antibody responses against antigenically distinct swine and human IAVs. Mismatched vaccination regimens not only failed to confer clinical and virological protection post-challenge but also exacerbated disease and pathology. In particular, heterologous-boosted pigs showed prolonged clinical disease and increased pulmonary pathology compared to mock-vaccinated pigs. Our results demonstrated that H1-specific heterologous prime-boost vaccination, rather than enhancing cross-protection, worsened the clinical outcome and pathology after challenge with the antigenically distant A/swine/Georgia/27480/2019 strain.

## Introduction

Influenza A viruses (IAVs) are considered important respiratory viral pathogens of both human and animals, causing substantial mortality and high economic losses. Influenza disease in swine and humans appears as an acute high-morbidity and low-mortality respiratory tract infection often characterized by anorexia, short-term fever, coughing, and breathing difficulties (1–3). Influenza subtypes that are considered to be originally spilled over from humans into swine through reverse-zoonotic events, namely, the H1N1, H1N2, and H3N2 variants, are currently endemic in swine (4, 5). Although zoonotic and reverse-zoonotic infections are considered to be sporadic, the bidirectional transmission dynamics of influenza viruses contribute to the ever-increasing antigenic diversity and facilitate the potential of the emergence of a novel pandemic virus (6, 7).

The epidemiology of IAV in the North American swine populations over the last 30 years is quite complex due to the rapid genetic evolution and immense antigenic variation, which are facilitated by frequent antigenic drift and shift events (7, 8). While the alpha clade (1A.1.1.) or “classical” swine H1N1 viruses, which evolved from the Spanish flu 1918 pandemic strain, remained relatively genetically and antigenically stable in the US swine for approximately 70 years, the landscape of swine IAVs (swIAV) was dramatically changed by the emergence of a novel triple-reassortant H3N2 virus in 1998 (9, 10). This H3N2 virus that contained the swine–human–avian triple-reassortant internal gene (TRIG) cassette reassorted with the circulating previously established alpha clade H1N1 viruses, resulting in the formation of the beta clade (1A.2) and gamma clade (1A.3.3.3) lineages of H1N1 swIAVs (11, 12).

During the early 2000s, following two separate introductions of human seasonal H1N1 viruses into swine, these human-origin viruses reassorted with the endemic strains at the time and resulted in the generation of the novel delta-clade swIAVs, which were further subclassified into delta1 (1B.2.2) and delta2 (1B.2.1) clades (10, 12). In contrast to alpha, beta, and gamma clade swIAVs, delta-H1 viruses are composed of a hemagglutinin (HA) of human seasonal influenza virus origin (13). Finally, the emergence of the 2009 pandemic H1N1 strain in humans and its subsequent spread into swine populations resulted in extensive reassortment events that drove the epidemiology of North American swIAVs to become even more complicated (10). The 2009 pandemic virus H1 (1A.3.3.2), while mostly related to the gamma clade viruses circulating in the US swine herds at that time, established a separate unique subclade within the gamma phylogenetic clade (10). In addition to the antigenic shift events, residual accumulation of point mutations on the external viral proteins, HA and neuraminidase (NA), over time resulted in further diversification of endemic influenza viruses in the US swine populations (7, 14).

Developing effective prophylactic influenza vaccination strategies for both humans and animals is pivotal in controlling the disease. While several novel broadly protective vaccine approaches have been developed and tested for both human and animal health, multivalent whole-inactivated virus (WIV) vaccines are the most commonly used vaccine platform globally (15). Numerous studies have demonstrated that although commercially available inactivated IAV vaccines elicit adequate protection against homologous viruses both in swine and in humans, they often fail to establish sufficient cross-protection against antigenically divergent homosubtypic and heterosubtypic viruses (16, 17). Thus, given the vast antigenic diversity of viruses currently circulating in US swine populations, vaccination can only be used as a narrow measure aimed to control the disease burden. The most significant shortcomings of inactivated flu vaccines are that they elicit predominantly strain-specific serological responses primarily targeting the immunodominant globular head of the HA protein that decline rapidly following booster vaccination and that they fail to induce mucosal immunity and strong cell-mediated immune protection (18, 19).

A vaccination strategy that has been recently explored and has been shown to broaden the antibody responses of experimental or commercial WIV influenza vaccines is heterologous prime-boost vaccination, i.e., sequential immunization with antigenically mismatched homosubtypic vaccine strains (20–25). In the current study, we utilized two H1N1 WIV vaccine strains, alpha clade A/swine/Minnesota/A02636116/2021 (1A.1.1) and pandemic-clade A/California/07/2009 (1A.3.3.2), mismatched to the challenge delta2 clade A/swine/Georgia/27480/2019 H1N2 virus (1B.2.1). The main objectives of this study were to examine the immunogenicity and protective efficacy of diverse mismatched WIV H1 vaccination strategies, including monovalent, bivalent, and heterologous prime-boost vaccination regimens, based on the three distinct US swine H1 lineages, against antigenically distinct homosubtypic US swIAVs. We report that H1 heterologous prime-boost vaccination, rather than enhancing cross-protection, exacerbated clinical disease and pathology following challenge with a virus antigenically distinct to the vaccine viruses strain.

## Materials and methods

### Cells and virus stocks

Madlin-Darby canine kidney (MDCK) cells (ATCC CCL 34, American Type Culture Collection, Manassas, VA, USA) were maintained in Dulbecco’s modified Eagle’s media (DMEM) (Corning Life Sciences, Corning, NY, USA) and medium was supplemented with 10% fetal bovine serum (GE Healthcare Life Sciences, Westborough, MA, USA), 1% antibiotic–antimycotic solution (Corning Life Sciences, Corning, NY, USA), in a 37°C incubator with 5% CO_2._ IAV strains that were used for vaccine and challenge purposes were propagated in MDCK cells. For swine vaccination, we utilized three swIAV strains: A/California/07/2009 H1N1 virus (CA09), which contains a pandemic (pdm)-clade HA; A/swine/Minnesota/A02636116/2021 H1N1 virus (MN21), which contains an alpha clade HA; and A/swine/Georgia/27480/2019 H1N2 virus (GA19), a swine IAV field isolate, which contains a delta2 clade HA. GA19 was also used for challenge. These strains were propagated in MDCK cells. The virus stock that was used for challenge was stored at −80°C and the virus titer [50% median tissue culture infectious dose (TCID50)] was calculated using the Reed and Muench formula (26).

### Animals

Thirty-nine 7-week-old influenza virus-naïve and porcine reproductive and respiratory syndrome virus-naive conventional cross-bred Yorkshire/Hampshire male and female pigs were obtained from Auburn University’s Swine Research Center (influenza virus and porcine reproductive and respiratory syndrome virus-seronegative farm). All study procedures were reviewed and approved by the Institutional Animal Care and Use Committee (IACUC) of Auburn University, under IACUC Protocol 2022-4095. Upon arrival (1 week prior to vaccination), animals were housed in the BSL-2 facilities of the Sugg Laboratory for Animal Health Research and were prophylactically treated with enrofloxacin (Baytril, Elanco, Indianapolis, IN) and ceftiofur crystalline free acid (Excede, Zoetis Inc., Kalamazoo, MI) according to the manufacturer’s instructions. Pigs were randomly divided into six groups of six animals and one group of three animals, as outlined in the *Swine vaccination, virus challenge, and sample collection* section. Each experimental group was housed in a separate isolation unit with a HEPA air filtration system. Animals were acclimated for 1 week prior to the start of the study. Food and water were provided *ad libitum*.

### Vaccine preparation

Swine influenza vaccine strains (CA09, MN21, and GA19 viruses) grown in MDCK cells were inactivated by β-propiolactone (Sigma-Aldrich, St. Luis, MO) treatment as previously described (27). Sufficient virus inactivation was demonstrated by failure of virus replication in two-serial passages in MDCK cells (28). Following inactivation, vaccine preparations were formulated with a commercial mineral oil-in-water (O/W) emulsion (Montanide™ ISA 15 VG), which served as the adjuvant solution. WIV vaccine formulations were mixed at a volume-to-volume ratio of 85:15 virus suspension to adjuvant, prepared under a low shear rate, and stored at 4°C according to the manufacturer’s instructions, 1 day prior to each vaccination. Monovalent vaccine formulations (CA09, MN21, and GA19) contained prior to adjuvant addition approximately 128 hemagglutination units (HAU)/mL of WIV while bivalent vaccine formulations (CA09-MN21) contained approximately 256 HAU/mL (128 HAU of each WIV). In assessing the HAU of the vaccine virus stocks, we conducted a hemagglutination assay for each virus, as previously described (29). After calculating the HA units within the stock, a subsequent dilution was performed, to achieve a final HA concentration of either 128 or 256 HA units/mL.

### Swine vaccination, virus challenge, and sample collection

Pigs were randomly assigned to six groups of six animals and one group of three animals. Pigs were prime-vaccinated at the age of 8 weeks and booster immunization was administered 3 weeks later, at the age of 11 weeks. Animals were vaccinated with either the monovalent CA09, MN21, and GA19 WIV vaccines, the bivalent CA09-MN21 WIV vaccine, or a phosphate-buffered saline (PBS) (Corning Life Sciences, Corning, NY, USA) in adjuvant solution. Each pig received a deep intramuscular injection into the neck with 1 mL of the monovalent or bivalent WIV vaccine or 1 mL of PBS-O/W adjuvant solution. Three homologous prime-boost vaccine groups (*n* = 6) were each vaccinated with the monovalent CA09, MN21, and GA19 WIV vaccine. The heterologous prime-boost vaccine group (*n* = 6) was prime-vaccinated with the monovalent CA09 WIV vaccine and was boosted on day 21 post-prime (pp) with the monovalent MN21 WIV vaccine. The bivalent vaccine group (*n* = 6) was both prime and boost vaccinated with the bivalent CA09-MN21 WIV vaccine. Control groups included a mock-vaccinated/challenged group (MV/C) (*n* = 6) and a mock-vaccinated/non-challenged group (MV/NC) (*n* = 3). Four weeks after boost vaccination, all pigs except the MV/NC control group were challenged with the GA19 H1N2 virus. On the day of challenge, GA19 virus was prepared for an inoculum of 10^5^ TCID50/mL. Swine were inoculated with 4 mL total volume, split into 2 mL intranasally (IN) (1 mL per nostril) using a mucosal atomization device (MAD Nasal™ Atomization Device, Teleflex, Wayne, PA) and 2 mL intratracheally (IT) (2 mL of 10^5^ TCID_50_/mL per route). Animals were clinically scored daily post-challenge (pc) based on rectal temperature, respiratory rate, and assessment of clinical behavior. Specifically, rectal temperature scores, measured using digital thermometers, ranged from 0 to 3 (<39.4°C = 0, 39.4–39.9°C = 1, 40–40.5°C = 2, >40.6°C = 3); respiratory rate scores assigned per minute ranged from 0 to 2 (20–40/min = 0, 41–59/min = 1, >60/min = 2); and clinical behavior score, based on coughing (absent = 0, present = 1) and depression (absent = 0, present = 2), ranged from 0 to 3, as previously described (30). To assess virus shedding, nasal swabs were collected daily starting 2 days prior to inoculation until day 5 pc, as previously described (31). All challenged animals were humanely euthanized with a lethal dose of pentobarbital (intravenous administration) on day 5 pc. MV/NC animals were necropsied prior to challenge at day 49 pp. At necropsy, bronchoalveolar lavage fluid (BALf) was collected for cytopathology, and respiratory tissue samples, including nasal turbinates, trachea, right lung lobes, and left lung lobes, were harvested for further virological and histopathological evaluation. The experimental design of the vaccination-challenge study is summarized in **Table 1**.

**Table 1:** Experimental design of vaccination-challenge study.

Groups	Number of Animals	Vaccine Type	Prime (Day 0 pp)	Boost (Day 21 pp)	Challenge (Day 51 pp)
MV/NC	3	–	ISA 15	ISA 15	–
MV/C	6	–	ISA 15	ISA 15	GA19 (H1N2)
mono GA19 (×2)	6	Monovalent	GA19 WIV (H1N2)	GA19 WIV (H1N2)	GA19 (H1N2)
mono MN21 (×2)	6	Monovalent	MN21 WIV (H1N1)	MN21 WIV (H1N1)	GA19 (H1N2)
mono CA09 (×2)	6	Monovalent	CA09 WIV (H1N1)	CA09 WIV (H1N1)	GA19 (H1N2)
biv CA09-MN21 (×2)	6	Bivalent	CA09 WIV (H1N1) + MN21 WIV (H1N1)	CA09 WIV (H1N1) + MN21 WIV (H1N1)	GA19 (H1N2)
mono CA09 → mono MN21	6	Monovalent Heterologous Prime-Boost	CA09 WIV (H1N1)	MN21 WIV (H1N1)	GA19 (H1N2)

MV/NC, mock-vaccinated/non-challenged; MV/C, mock-vaccinated/challenged; mono, monovalent; biv, bivalent; GA19, A/swine/Georgia/27480/2019; MN21, A/swine/Minnesota/A02636116/2021; CA09, A/California/07/2009; WIV, whole-inactivated virus; ISA 15, commercial oil-in-water adjuvant emulsion; pp, post-prime.

### Hemagglutination inhibition assay

Serum samples were collected post-prime vaccination (day 0 pp), at boost vaccination (day 21 pp), at 2 weeks post-boost (day 35 pp), at 4 weeks post-boost (day 49 pp) prior to the challenge, and at day 5 pc. Sera for hemagglutination inhibition (HAI) titers were processed according to the WHO protocol and as previously described (32). Briefly, serum samples were treated overnight at 37°C with a receptor-destroying enzyme (RDE) (Denka Seiken Co Ltd) to remove nonspecific inhibitors. Following overnight treatment, sodium citrate (1.5%) was added to the samples and the mixtures were heated at 56°C for 30 min to inactivate the residual RDE and complement factors. RDE-pretreated sera were incubated with packed 50% turkey red blood cells (RBCs) for 1 h at 4°C, to remove any non-specific cryoglobulins interfering with RBC and antigen–antibody binding. Following centrifugation at 10,000 *g* for 3 min at 4°C, the supernatant (HAI-treated sera) was collected, aliquoted, and stored at −23°C for further use in HAI assays.

The following steps were carried out at room temperature. Twofold serially diluted HAI-treated sera (50 μL/well) were incubated with 4 HA units of MDCK-grown swine or human IAVs that are listed in **Supplementary Table 1** for 1 h. Next, 50 μL of 0.5% turkey RBCs were added to each well and hemagglutination inhibition titers were recorded after 45–60 min (33, 34). North American swine IAV strains employed in the HAI assay that were not held in our repository were obtained from the U.S. Department of Agriculture (USDA) swine influenza repository at the National Veterinary Service Laboratories. Sample analysis was performed in duplicates and the HAI antibody titers were expressed as the reciprocal of the highest dilution of serum that conferred inhibition of hemagglutination.

### Nasal swab reverse transcription polymerase chain reaction assay

To assess virus shedding, virus in nasal swabs collected daily post-challenge from each animal was suspended in 2 mL of PBS supplemented with 1% antibiotic–antimycotic solution by mechanical agitation for 10 min at 4°C and stored as aliquots at −80°C until RNA isolation for reverse transcription polymerase chain reaction (RT-PCR) assays. Total RNA was extracted from 200 µL of nasal swab samples harvested at necropsy using RNAzol-RT (Molecular Research Center, Cincinnati, OH - CAT #RN 190) according to the manufacturer’s instructions and resuspended in 20 µL. An RT-PCR reaction was performed using 2 μL of extracted total RNA, 6.25 μL of Taqman^®^ Fast Virus 1-Step Master Mix (Life Technologies- CAT # 4444434), 0.5 μL of forward primer (20 nmol) (5’-AGA TGA GTC TTC TAA CCG AGG TCG-3’, Biosearch Technologies, Cat# INFA-F-20), 0.5 μL of reverse primer (20 nmol) (5’-TGC AAA AAC ATC TTC AAG TCT CTG-3’, Biosearch Technologies, Cat# INFA-R-20), 1 μL of probe (5 nmol) (FAM-TCA GGC CCC CTC AAA GCC GA-BHQ, Biosearch Technologies, Cat# INFA-P-5), and 14.75 μL of HyPure™ Molecular Biology Grade Water (GE Healthcare Life Sciences, HyClone Laboratories, Logan, UT) in a total volume of 25 μL per reaction. Thermal cycling was performed under the following conditions: reverse transcription for 20 min at 50°C, inactivation of reverse transcriptase for 10 min at 95°C, followed by 45 cycles of denaturation at 95°C for 10 seconds, and annealing/amplification at 55°C for 20 s. A standard curve was generated by using a 10-fold dilution series from the A/swine/Georgia/27480/2019 virus stock that was used for animal inoculation and was of known TCID_50_ titer (10^7^ TCID_50_). Based on the standard curve, CT values from the extracted viral RNA of nasal swab samples were converted to Relative Equivalent Units (REU) as previously reported (35).

### Virus titration (TCID_50_) assay

To compare virus replication in the upper and lower respiratory tract between vaccinated and mock-vaccinated challenged animals, we performed virus titration assays on respiratory tissue homogenates. Specifically, respiratory tissue samples (nasal turbinates, trachea, right lung lobes including accessory lung lobe, and left lung lobes) harvested after euthanasia were homogenized prior to virological evaluation, as previously described (30). Briefly, respiratory tissue samples were weighed to achieve a 10% weight (g)-to-volume (w/v) of tissue suspended in PBS. Tissue suspensions were then homogenized and centrifuged. Following centrifugation, tissue homogenate supernatants were collected, aliquoted, and stored at −80°C for TCID_50_ assays.

For the virus titration assay, following serial 10-fold dilution, tissue homogenate samples were used to inoculate confluent MDCK cell monolayers in serum-free media. After 72-h incubation at 37°C, wells were evaluated for the presence of cytopathogenic effect (CPE) and virus titers (TCID_50_) were determined by the Reed–Muench method (26).

### Cytopathological evaluation of bronchoalveolar lavage fluid

At necropsy time points (day 49 pp for MV/NC and day 5 pc for all other groups), lungs were harvested from all the animals (*n* = 39) for virological and histopathological evaluation and BALf collection. BALf samples were processed as previously described (30). Briefly, following the removal of the pluck, the lungs were washed with 50 mL of Ca/Mg-deficient PBS (Corning Life Sciences, Corning, NY, USA). Upon collection, the samples were passed through a 70-µm pore size cell strainer (VWR International, Radnor, PA) to remove mucus plugs, and then centrifuged at 1200 g for 5 min. Subsequently, the BALf was treated with 1× Lysis Buffer (BioLegend, San Diego, CA) for 5 min to lyse RBCs. Following centrifugation, pelleted BALf cells were counted (Countess II Automated Cell Counter, Thermo Fisher Scientific, Carlsbad, CA) and resuspended with the appropriate volume of PBS to achieve a final cell density of 0.5–1 × 10^5^ cells. The cells were stained with a three-step Wright-Giemsa Stain (Quick Stain, VWR International, Radnor, PA) and the BALf cell populations (macrophages, neutrophils, and lymphocytes) were expressed as the percentage of the total number of cells counted (36). BALf cytology slides were examined by two individuals blinded to treatment groups, including a board-certified veterinary clinical pathologist and the cell counts were averaged.

### Histopathologic evaluation of pulmonary tissues

Following necropsy, gross lung lesions were assessed to determine the macroscopic pulmonary pathology profiles between the different groups. Additionally, lung tissue samples (from the most prominently affected right and left lung lobes) were collected, fixed in 10% neutral buffered formalin, sectioned, and stained with hematoxylin and eosin stains (H&E) for microscopic evaluation (Olympus BX53; Olympus Scientific Solutions Technologies Inc., Waltham, MA). Lung sections were examined by two individuals blinded to treatment groups, including a board-certified veterinary anatomic pathologist and the scores were averaged. Histopathology scores considered five parameters to reflect the severity of pulmonary pathology following influenza infection, as previously described (30). Specifically, pulmonary tissues were scored on a 17-point scale based on the composite scores of five parameters: (i) the percent of lung section affected, (ii) the extent of bronchial and bronchiolar epithelial changes, (iii) the severity of interstitial pneumonia, (iv) the degree of lymphocytic peribronchiolar cuffing, and (v) Bronchus-associated lymphoid tissue (BALT) hyperplasia, as previously described with some modifications (30, 37). The first three parameters (i–iii) were scored from 0 to 4, with 4 being the most severe; parameter (iv) was scored from 0 to 3, with 3 being the most severe; and the last parameter (v) was scored from 0 to 2, with 2 being the most severe.

### Statistical analysis

GraphPad Prism software (version 9, San Diego, CA, USA) was used for statistical analysis. Statistical comparisons between vaccinated groups were performed via one-way ANOVA with *post-hoc* Bonferroni multiple comparisons test for the comparison of virus load in nasal turbinates and left lung homogenates and composite, right lung, and left lung pathology scores between groups with an alpha of 0.05 (*p* ≤ 0.05). A two-way ANOVA with *post-hoc* Tukey’s multiple comparison test was used for the comparison of HAI titers, clinical scores, and nasal swab virus titers and Dunnett’s *post-hoc* multiple comparisons test for BALf cytology between groups with an alpha of 0.05 (*p* < 0.05). The nonparametric Kruskal–Wallis test was used to test differences between groups for the trachea and right lung virus load.

## Results

### Heterologous prime-boost vaccination did not augment HAI antibody titers against vaccine constituents compared to homologous monovalent and bivalent prime-boost vaccination regimens

For vaccination purposes, we used three antigenically distinct contemporary US H1 swine influenza A viruses (swIAVs): an H1N1 swIAV [A/swine/Minnesota/A02636116/2021 (MN21)], which has a classical alpha-clade HA; an H1N1 swIAV [A/swine/California/07/2009 (CA09)], which has an HA of 2009 pandemic (pdm) origin; and an H1N2 swIAV [A/swine/Georgia/27480/2019 (GA19)], which has a human-like delta2 clade HA. The phylogenetic relationships of the HA genome segment between the vaccine viruses is shown in **Supplementary Figure 1**. While all three vaccine viruses are homosubtypic, the amino acid homology in their HA is quite variable as shown in **Supplementary Table 1**.

**Figure 1:**
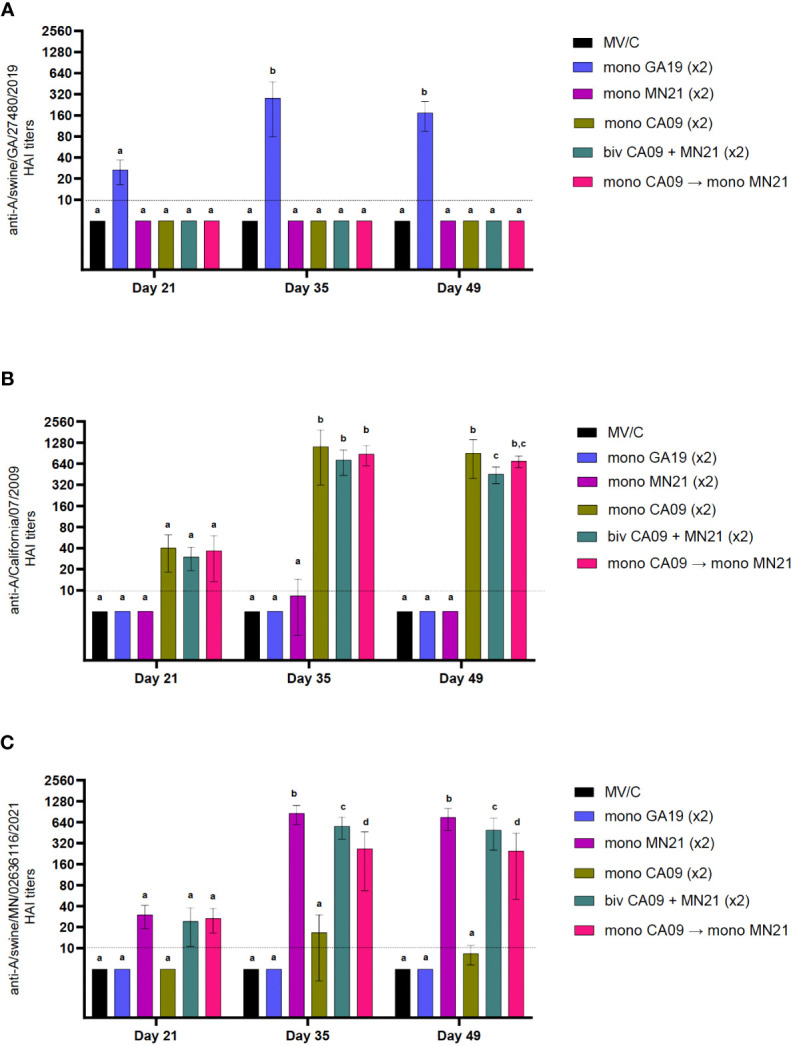
Heterologous prime-boost vaccination did not augment HAI antibody titers against vaccine constituents compared to homologous monovalent and bivalent prime-boost vaccination regimens. Five groups of pigs (n = 6) were vaccinated in a homologous or a heterologous prime-boost regimen 3 weeks apart at day 0 and day 21. Serum samples from the groups were assessed on day 21 at boost vaccination, day 35, and day 49 pp (day −2 pre-challenge) for hemagglutination inhibition titers against **(A)** A/swine/Georgia/27480/2019 H1N2, **(B)** A/California/07/2009 H1N1, and **(C)** A/swine/Minnesota/A02636116/2021 H1N1. Antibody titers are compared between homologous-boosted monovalent GA19 (blue), MN21 (purple), and CA09 (olive) groups, the bivalent CA09+MN21 vaccine group (cyan), and the heterologous prime-boosted vaccine group (crimson red). Bars represent geometric mean HAI titers and error bars represent 95% confidence interval (CI). Statistical analysis was performed by two-way ANOVA test. Statistically significant differences (p < 0.05) between vaccine group geometric means are noted by different lowercase letters.

We vaccinated pigs at a 21-day interval with a homologous prime-boost vaccination regimen with either one of three monovalent WIV vaccines (GA19, MN21, and CA09) or a bivalent vaccine composed of the CA09 and MN21 whole inactivated viruses. Additionally, a fifth group of pigs was prime immunized with the CA09 monovalent WIV vaccine and boosted with the MN21 monovalent WIV vaccine (heterologous-boosted group). Serum was collected at days 0, 21, 35, and 49 pp vaccination. Anti-HA antibody titers were determined by performing HAI assay. Our first goal was to assess the HAI titers against the three vaccine viruses GA19, MN21, and CA09 (**Figure 1**). According to previous literature, we consider HAI titers above 1:40 to be seroprotective (38–41).

Independent of the vaccination strategy used, prior to prime immunization, all groups were seronegative for IAV. As expected, MV/C pigs did not show measurable HAI antibody titers at any time point assayed prior to the challenge. Notably, throughout the study vaccinated pigs demonstrated minimal or no detectable cross-reactive HAI titers against viruses that were not included in their respective vaccine formula (**Figure 1**). At day 21 pp, after a single vaccine dose, all vaccination regimens induced HAI antibodies below the positive cutoff reciprocal titer of 1:40 against the corresponding homologous viruses (**Figure 1**). However, peak HAI antibody titers for all five vaccine groups were detected at 2 weeks post-boost against viruses included in the original vaccine. Homologous prime-boost monovalent WIV vaccines CA09, MN21, and GA19 generated approximately 25-fold, 28-fold, and 9-fold increases in HAI titers, respectively, from day 21 pp, and these titers were found to be significantly higher than in MV/C pigs (*p* < 0.0001). This trend continued at day 49 pp, with sera from all monovalent vaccinates showing elevated HAI titers compared to MV/C pigs (*p* < 0.0001), although sustaining a mild decrease in their antibody titers of up to a half-log lower compared to day 35 pp (**Figure 1**).

While both the bivalent and prime-boosted groups generated comparable HAI titers to the monovalent CA09 group against the A/swine/California/07/2009 virus at day 35 pp, 2 weeks later (day 49 pp), antibody titers were found to be significantly lower in the bivalent group compared to CA09 vaccinates (*p* < 0.05). At that point, we detected no significant differences in the anti-CA09 antibody titer between the heterologous prime-boost group and either the bivalent or monovalent CA09 vaccine group (**Figure 1B**). On the other hand, at day 35 pp, anti-MN21 HAI titers were significantly higher in the MN21 group compared to both the bivalent and heterologous prime-boosted groups (*p* < 0.05 and *p* < 0.0001) with the former group demonstrating significantly elevated titers compared to the latter (*p* < 0.05). Antibody titers against A/swine/Minnesota/A02636116/2021 maintained a similar pattern at day 49 pp (**Figure 1C**).

Overall, all three vaccination regimens induced seroprotective titers against viruses included in the vaccine formulation following two vaccine doses. However, these data demonstrate that monovalent vaccination conferred the highest mean HAI antibody titers against the respective homologous viruses as compared to both the bivalent and heterologous prime-boost vaccination.

### Heterologous prime-boosting did not expand cross-reactive HAI antibody responses against antigenically distinct swine and human IAVs

Our next goal was to compare the different vaccination approaches deployed in our study in their ability to broaden the immunological HAI cross-reactivity against antigenically distinct swine and human IAVs. In this regard, we included in our assay 15 contemporary representative H1-specific swine IAVs (including the three vaccine viruses) of the main North American swIAV phylogenetic clades that were circulating in US swine populations over the past decade. According to Bakre and colleagues, the four dominant HA phylogenetic clades of US H1 swIAVs are gamma (1A.3.3.3) and delta 2 (1B.2.1) clade viruses followed by the pandemic (1A.3.3.3) and alpha (1A.1.1) clade viruses (42). In addition to swIAVs, we examined HAI responses against six representative human IAVs isolated over the last century. The HA amino acid homology and phylogenetic relationship of the viruses employed in this assay to the vaccine strains employed in our study are presented in **Supplementary Table 1** and **Supplementary Figure 1**, respectively.

For the purpose of this assay, we tested sera collected at 2 weeks post-boost as it was demonstrated that peak antibody titers against the homologous viruses were achieved at day 35 pp, shown in **Figure 1**. As expected, sera from mock-vaccinated animals did not demonstrate any appreciable HAI titers measured at day 35 pp (results not shown). Previous studies have demonstrated that there is limited to no HAI cross-reactivity between viruses that belong to phylogenetically distinct swIAV clades (43–46). Indeed, regardless of the WIV strains used, monovalent vaccination, with a few exceptions, failed to elicit cross-reactive responses against the heterologous vaccine viruses and viruses belonging to antigenically distant lineages to their corresponding WIV vaccine constituent(s) (**Table 2**).

**Table 2:** Heterologous prime-boosting did not expand cross-reactive HAI antibody responses against antigenically distinct swine and human IAVs.

		Vaccine Groups				
Tested Viruses	Designated Clade/Global Nomenclature	mono GA19 (delta2)	mono MN21 (alpha)	mono CA09 (pdm)	biv CA09+MN21 (pdm +alpha)	mono CA09 (pdm) → mono MN21 (alpha)
**A/swine/Minnesota/A02636116/2021/H1N1**	**alpha/1A.1.1**	**<10**	**853**	**17**	**560**	**267**
**A/swine/North Carolina/A02246984/2021/H1N1**	**alpha/1A.1.1**	**<10**	**620**	**11**	**333**	**213**
**A/swine/South Dakota/A01823237/2015/H1N2**	**alpha/1A.1.1**	**<10**	**427**	**<10**	**240**	**103**
**A/swine/Kansas/A01785470/2018/H1N1**	**beta/1A.2**	**<10**	**12**	**27**	**19**	**17**
**A/swine/Missouri/A01432837/2013/H1N2**	**beta/1A.2**	**<10**	**18**	**50**	**32**	**28**
**A/swine/Indiana/A01732425/2016/H1N1**	**gamma/1A.3.3.3**	**<10**	**<10**	**73**	**40**	**53**
**A/swine/North Carolina/A02245704/2020/H1N1**	**gamma/1A.3.3.3**	**<10**	**<10**	**34**	**18**	**17**
**A/swine/South Dakota/A01349306/2013/H1N1**	**gamma2/1A.3.2**	**<10**	**<10**	**88**	**27**	**73**
**A/California/07/2009/H1N1**	**pdm/1A.3.3.2**	**<10**	**10**	**1120**	**720**	**880**
**A/swine/Colorado/A02635828/2021/H1N1**	**pdm/1A.3.3.2**	**<10**	**<10**	**667**	**427**	**533**
**A/swine/Oklahoma/A01290605/2013/H1N1**	**delta1b/1B.2.2.1**	**22**	**<10**	**<10**	**<10**	**11**
**A/swine/Iowa/A02431617/2019/H1N1**	**delta1a/1B.2.2.2**	**57**	**<10**	**11**	**<10**	**<10**
**A/swine/Illinois/A02214842/2017/H1N2**	**delta2/1B.2.1**	**187**	**<10**	**<10**	**<10**	**<10**
**A/swine/North Carolina/A02245213/2019/H1N1**	**delta2/1B.2.1**	**147**	**<10**	**<10**	**<10**	**<10**
**A/swine/Georgia/27480/2019/H1N2**	**delta2/1B.2.1**	**280**	**<10**	**<10**	**<10**	**<10**
**A/Puerto Rico/8/1934/H1N1**	**delta-like/1B.2**	**21**	**15**	**10**	**11**	**<10**
**A/New Caledonia/20/1999/H1N1**	**delta-like/1B.2**	**77**	**<10**	**<10**	**<10**	**<10**
**A/Brisbane/59/2007/H1N1**	**delta-like/1B.2**	**83**	**<10**	**<10**	**<10**	**<10**
**A/Michigan/45/2015/H1N1**	**pdm/1A.3.3.2**	**<10**	**15**	**613**	**227**	**427**
**A/Michigan/272/2017/H1N1**	**pdm/1A.3.3.2**	**<10**	**<10**	**480**	**187**	**313**
**A/Wisconsin/588/2019/H1N1**	**pdm/1A.3.3.2**	**<10**	**<10**	**297**	**173**	**153**

mono, monovalent; biv, bivalent; GA19, A/swine/Georgia/27480/2019; MN21, A/swine/Minnesota/A02636116/2021; CA09, A/California/07/2009; pdm, pandemic.

Serum samples from all groups collected at day 35 pp (2-week post-boost) were assessed for hemagglutination inhibition antibody titers against a broad panel of representative swine and human influenza viruses isolated over the last century. The initial dilution of serum used for the HAI assay was 1:10. Each row represents the mean HAI titers per vaccine group against the corresponding IAV strain listed in the table. HAI titers above 40 are considered seroprotective. Mean antibody titers are color-coded based on levels of cross-reactivity.

HAI Antibody Titer: 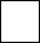
<40 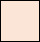
40–80 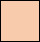
81–160 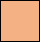
161–320 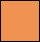
>320.

Specifically, the MN21 and GA19 vaccination elicited seroprotective antibody responses that were limited to swine alpha-clade viruses and delta-clade viruses and human delta-like viruses, respectively. Vaccination with the CA09 WIV, however, induced cross-reactive responses above the positive cutoff reciprocal HAI titer of 1:40, not only against homologous swine and human influenza viruses containing pdm-clade HA but also against two out of three gamma clade viruses and one beta clade virus tested in the assay. Although heterologous-boosting and bivalent vaccination increased the breadth of HAI responses compared to the CA09 and MN21 monovalent vaccines by mounting titers against viruses antigenically related to those included in the original formulation, they did not expand cross-reactivity against the antigenically distinct delta and delta-like clade viruses (**Table 2**). Additionally, the bivalent and heterologous-boosted vaccine groups failed to evoke seroprotective responses against either beta clade virus tested in the assay. Overall, peak HAI antibody titers against each individual virus were only demonstrated in vaccinated pigs immunized with the homologous monovalent prime-boost vaccination regimen, with bivalent and heterologous-boosting vaccination approaches often inducing antibody titers that were multifold lower.

These data suggest that while heterologous-boosting and bivalent vaccination enhanced the breadth (but not the magnitude) of seroprotective HAI responses against antigenically related viruses compared to monovalent vaccines, they failed to expand serological responses against sufficiently distant swine and human H1 flu strains.

### Bivalent and heterologous prime-boosting vaccination strategies enhanced and prolonged clinical disease following challenge with A/swine/Georgia/27480/2019

**Figure 2:**
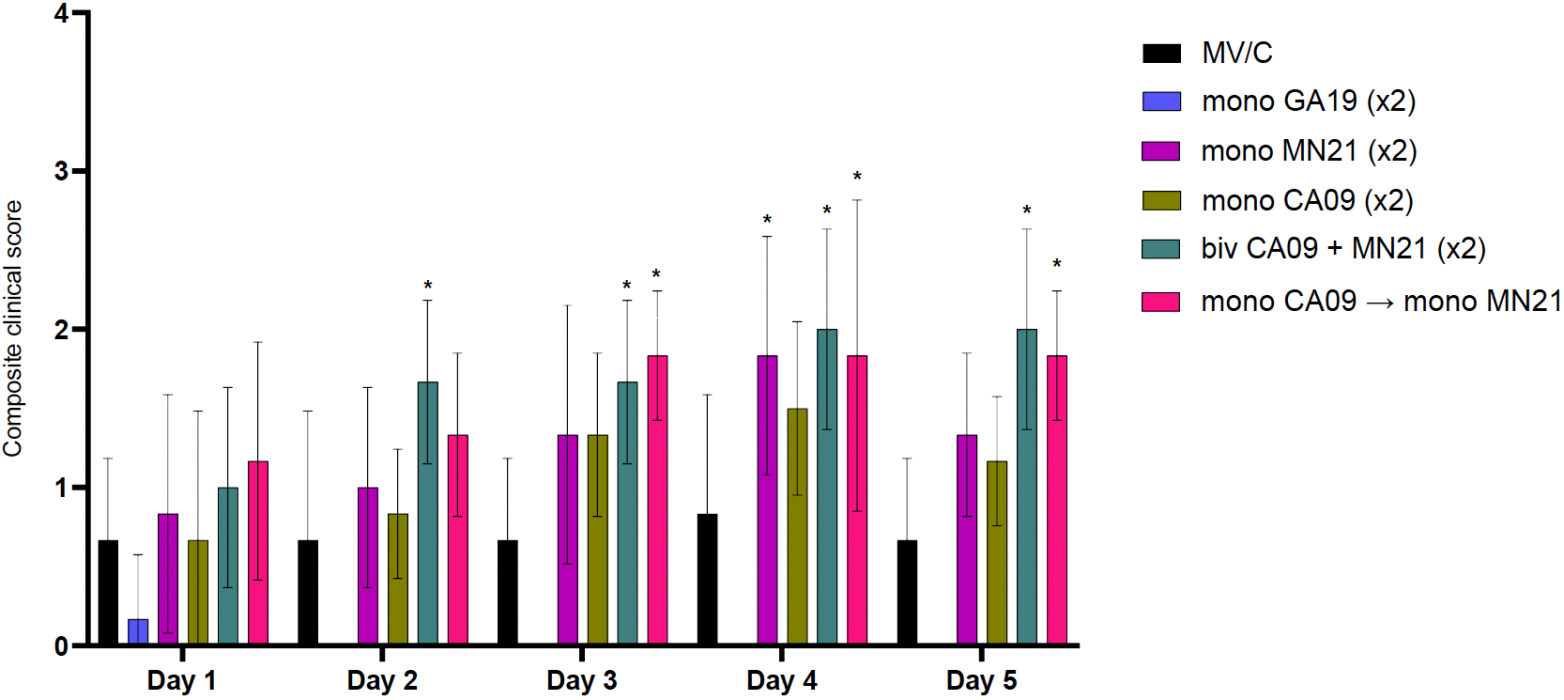
Bivalent and heterologous prime-boosting vaccination strategies enhanced and prolonged clinical disease following challenge with A/swine/Georgia/27480/2019. Clinical scores including rectal temperature, respiratory rate, and assessment of clinical demeanor (coughing and weakness/depression) were recorded daily following infection with the A/swine/Georgia/27480/2019 H1N2 challenge virus. Specifically, rectal temperature score ranged from 0 to 3 (<39.4°C = 0, 39.4–39.9°C = 1, 40–40.5°C = 2, >40.6°C = 3), respiratory rate per minute score ranged from 0 to 2 (20–40 = 0, 41–59 = 1, >60 = 2), and clinical behavior scores, based on coughing (absent = 0, present = 1) and depression (absent = 0, present = 2), ranged from 0 to 3. Bars represent the geometric mean of clinical scores and error bars denote 95% CI. Statistical analysis was performed by a two-way ANOVA test. Statistically significant differences (*p* < 0.05) in clinical scores’ means between MV/C and experimental vaccine groups per day are indicated by an asterisk (*).

Starting from day −2 prior to challenge through day 5 pc, rectal temperature, respiratory rate, and clinical signs, in the form of presence of coughing or weakness/depression, were recorded daily. Overall, clinical expression of influenza disease in MV/C was mild, with elevated rectal temperatures above normal detected in three pigs solely at day 4 pc. The delayed onset and mild course of clinical disease in the MV/C group could be attributed to the relatively low virus titer that was used in the inoculum (47). While mean clinical scores in the MN21 and CA09 vaccine groups were consistently higher compared to the MV/C group throughout the study, significant differences were only observed between MN21 and MV/C pigs at day 4 pc (*p* < 0.05) (**Figure 2**). Additionally, clinical disease in the bivalent and heterologous prime-boosted vaccine groups was more severe over a prolonged period of time in relation to all other challenged groups. Specifically, clinical scores were significantly increased in the heterologous prime-boosted and bivalent vaccine groups compared to MV/C control pigs over a period of three and four consecutive days, respectively (**Figure 2**). Respiratory distress was consistently observed in the majority of animals in these groups and soft coughing was noted at various times, particularly when pigs were aroused. Additionally, mild febrile responses were detected in the majority of the bivalent and heterologous prime-boosted vaccinates at days 3 and 4 pc. On the other hand, not unexpectedly, homologous GA19 vaccinated animals demonstrated minimal or no clinical signs following challenge with the identical virus (**Figure 2**).

Overall, our results demonstrate that bivalent and heterologous prime-boost immunization induced prolonged clinical disease in vaccinated swine compared to the MV/C group following challenge with the mismatched GA19 virus.

### Mismatched swine influenza vaccination elicited different inflammatory cytology profiles in BALf after influenza infection compared to the MV/C group

**Figure 3:**
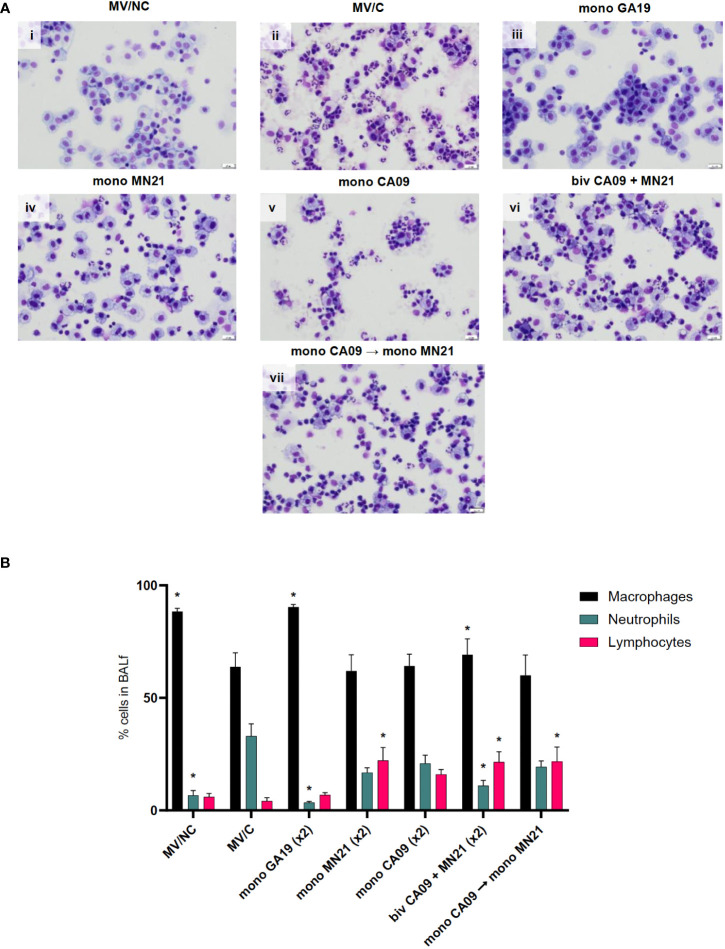
Mismatched swine influenza vaccination regimens elicited different inflammatory cytology profiles in BALf after influenza infection compared to the MV/C group. Bronchoalveolar lavage fluid (BALf) was harvested from lungs at day −2 pre-challenge for the MV/NC group and at day 5 pc from infected groups. **(A)** Concentrated cells were added to slides and stained with Wright-Giemsa stain and differential cell counts were determined microscopically. (i) MV/NC group, (ii) MV/C group, (iii) mono GA19 vaccine group, (iv) mono MN21 vaccine group, (v) mono CA09 vaccine group, (vi) bivalent CA09+MN21 bivalent vaccine group, and (vii) mono CA09 → mono MN21 heterologous prime-boosted vaccine group (20 μm scale bar, 40× magnification). **(B)** Cell counts were expressed as a percentage of total cells. Black bars represent the mean percentage of macrophages, cyan bars denote the mean percentage of neutrophils, and pink bars represent the mean percentage of lymphocytes in each experimental group. Error bars represent the standard error of the mean (SEM). Statistical analysis was performed by two-way ANOVA test. Statistically significant differences (*p* < 0.05) in population percentages between MV/C and experimental vaccine groups per cellular element are indicated by an asterisk (*).

To further characterize the vaccine-induced clinical protection, we harvested BALf from all animals at necropsy 5 days pc to assess the infiltration of inflammatory cells in the lower respiratory tract and their population dynamics following infection with the GA19 virus. In healthy MV/NC pigs, the BALf cytology profile was characterized primarily by tissue-resident macrophages along with the presence of a few non-degenerate neutrophils and rare small-sized lymphocytes (**Figure 3Ai**). Interestingly, while we observed minimal clinical signs in MV/C animals, their BALf cytology profile was characterized by mild to moderate BALf neutrophilic infiltration, which is a typical sign of uncomplicated influenza virus infection (**Figure 3Aii**). Homologous challenge following GA19 vaccination resulted in a similar cytology status to that of healthy MV/NC animals, with statistically significant lower percentages of neutrophils (*p* < 0.0001) and higher percentages of macrophages (*p* < 0.001) compared to the MV/C group (**Figures 3Aiii, B**).

In contrast, all four immunized with mismatched vaccine strain(s) to the challenge virus demonstrated mild to moderate lymphocytic pulmonary inflammation that was accompanied by a variable neutrophilic component that ranged from minimal in the bivalent vaccine group to mild to moderate in the MN21, CA09, and heterologous-boosted vaccine groups (**Figures 3Aiv–vii**). Regarding lymphocytic infiltration, the monovalent MN21, bivalent, and heterologous-boosted vaccine groups showed statistically significantly elevated percentages of lymphocytes in their BALf compared to MV/C animals (*p* < 0.05). While lymphocyte percentages trended higher in the monovalent CA09 vaccine group compared to MV/C, the difference did not reach statistical significance due to a large degree of inter-animal variability (**Figure 3B**).

In summary, these data suggest that mismatched vaccination favors the induction of different inflammatory cytology profiles in the BALf following challenge compared to non-vaccination, which are characterized by a primary lymphocytic or mixed lymphocytic–neutrophilic infiltration.

### Mismatched vaccination strategies failed to reduce virus shedding and replication in the respiratory tract

**Figure 4:**
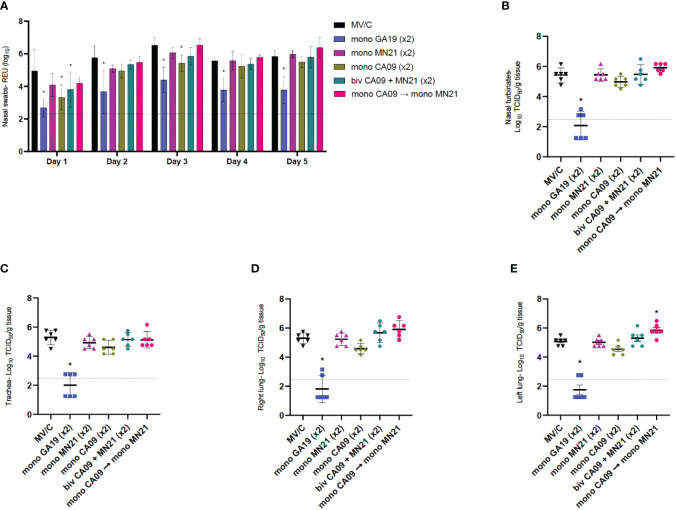
Mismatched vaccination strategies failed to reduce virus shedding and replication in the respiratory tract. Nasal swabs were collected from pigs daily post-challenge through euthanasia on day 5. Following necropsy, tissue samples from the respiratory tract including nasal turbinates, trachea, right lung lobes including the accessory lung lobe, and left lung lobes were harvested at day 5 post-challenge. Virus load in nasal swab samples was assessed by an RT-PCR assay. Titers were measured as relative equivalent units (REU) of RNA corresponding to a 10-fold dilution series of RNA extracted from infective MDCK culture medium at a 10^7^ TCID_50_ of the A/swine/Georgia/27480/2019 H1N2 challenge virus. Virus replication in the respiratory tract was determined via TCID_50_ assay in the respiratory tissue homogenates. **(A)** Mean viral titers from nasal swabs. **(B)** Mean viral titers of homogenized nasal turbinates. **(C)** Mean viral titers of homogenized trachea tissue. **(D)** Mean viral titers of right lung lobe tissue homogenates. **(E)** Mean viral titers of left lung lobe tissue homogenates. Black triangles represent MV/C pigs, blue squares represent GA19 vaccinated pigs, purple triangles represent MN21 vaccinated animals, olive rhombuses represent CA09 vaccinated pigs, cyan hexagons represent bivalent CA09+MN21 vaccinated pigs, and crimson red circles represent heterologous-boosted pigs. For A, bars represent mean virus titers and errors bars represent 95% CI. For B to E, horizontal lines represent the mean virus titer per homogenate for each group. Error bars represent 95% CI. Dotted lines indicate the limit of detection. Statistical analysis was performed by two-way ANOVA test **(A)**, one-way ANOVA test **(B, E)**, and Kruskal–Wallis test **(C, D)**. Statistically significant differences (*p* < 0.05) in mean virus titer between MV/C and experimental vaccine groups are indicated by an asterisk (*).

To assess virus shedding following challenge with the GA19 virus, we collected nasal swabs daily through day 5 pc. Titers were measured via an RT-PCR assay as relative equivalent units of RNA by employing a standard 10-fold dilution series of RNA extracted from the stock of the inoculation virus. Throughout the challenge, matched GA19 vaccinated pigs demonstrated statistically significant lower levels of mean virus shedding (*p* < 0.0001) that approached or were greater than two logs of virus load lower compared to MV/C pigs (**Figure 4A**). Overall, mismatched vaccination strategies failed to reduce virus shedding in pigs with the exception of day 1 pc where the bivalent and the monovalent CA09 vaccine groups showed significantly lower levels of viral RNA than mock-vaccinated animals (*p* < 0.001 and *p* < 0.0001, respectively). Additionally, we observed that CA09 vaccination reduced virus shedding at day 3 (*p* < 0.05) (**Figure 4A**). The monovalent MN21 and the heterologous prime-boosted vaccine groups consistently showed comparable virus shedding throughout the study to the MV/C pigs, with the virus load in the heterologous prime-boosted group trending higher than mock-vaccinated pigs from day 3 to day 5 (**Figure 4A**).

Next, we examined virus replication in the different compartments of the respiratory tract among the different experimental groups. Following tissue homogenization, viral titers were measured via a TCID_50_ assay. While GA19 vaccination induced almost complete protection against the homologous challenge virus in all four respiratory tissue sections (*p* < 0.0001), mismatched vaccination strategies did not offer any protection against virus replication of the GA19 virus (**Figures 4B–E**). The mean viral load in nasal turbinate, trachea, and right lung tissue homogenate samples was found to be comparable between mismatched vaccinated animals and MV/C pigs. While no significant differences were observed with the MV/C group in these homogenates, the bivalent and heterologous prime-boost vaccinated groups exhibited the highest virus titers among the mismatched vaccinated groups (**Figures 4B–E**). Interestingly, we noted that heterologous prime-boost vaccination increased pulmonary virus replication resulting in a statistically significant increase of virus load in left lung tissue homogenates compared to the MV/C group (*p* < 0.05), while in right lung homogenates, virus titer differences approached but did not reach statistical significance (*p* = 0.076) (**Figures 4D, E**).

Overall, we observed that while matched vaccination with the homologous virus offered almost sterilizing protection against virus shedding and replication in the respiratory tract, the mismatched vaccination strategies and particularly heterologous prime-boosting not only were unsuccessful in controlling the infection but additionally demonstrated the potential of enhancing pulmonary virus replication after challenge with A/swine/Georgia/27480/2019.

### Heterologous prime-boosting exacerbated pulmonary pathology following challenge with the A/swine/Georgia/27480/2019

Our next goal was to examine mismatched H1N1 vaccination strategies employed in our study in preventing the development of pulmonary pathology after challenge with the antigenically distinct A/swine/Georgia/27480/2019 H1N2 virus. First, we grossly examined the lungs for the assessment of macroscopic pulmonary lesion profiles. Regardless of the immunization regimen used, the majority of mismatched vaccinated animals demonstrated multifocal to coalescing to lobular dark red discoloration and, to a lesser extent, consolidation in the hilar and cranioventral regions that ranged from approximately 5% to 30% lung involvement. Mock-vaccinated challenged pigs demonstrated minimal to mild gross lesions that represented less than 5%–10% lung involvement. This is consistent with what has been previously observed following low virus titer inoculation in swine (47, 48).

**Figure 5:**
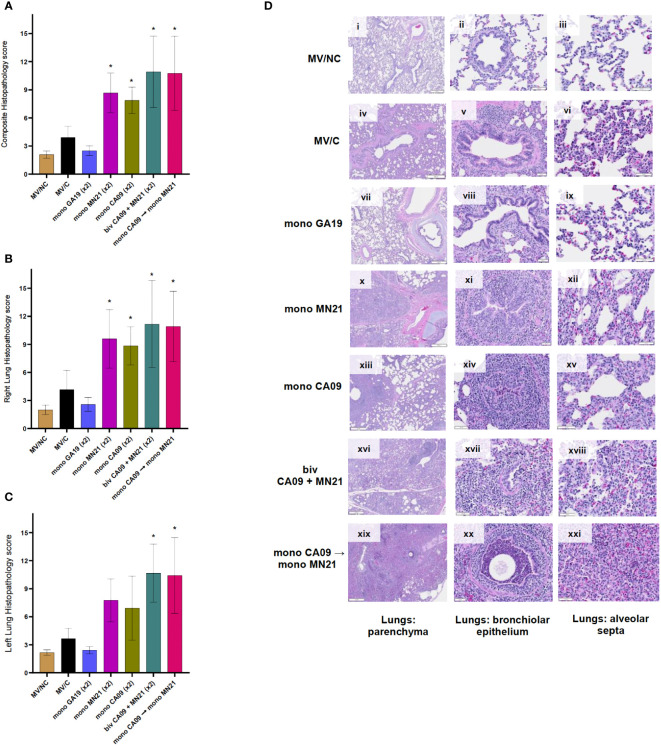
Heterologous prime-boosting exacerbated pulmonary pathology following challenge with A/swine/Georgia/27480/2019. Lung tissues were collected at necropsy 5 days post-challenge and were fixed in formalin. Five-micrometer sections were stained with H&E and examined by light microscopy. Lung sections were scored by two individuals including a board-certified veterinary pathologist. **(A)** Composite pulmonary histopathology scores from each experimental group. **(B)** Right lung mean histopathology scores. **(C)** Left lung mean histopathology scores. Bars represent mean histopathology scores and errors bars represent 95% CI. Statistical analysis was performed by one-way ANOVA test. Statistically significant differences (*p* < 0.05) in mean histopathology score with MV/C and experimental vaccine groups are indicated by an asterisk (*). **(D)** H&E-stained representative lung tissue sections from each experimental group. Parenchyma, 500 μm scale bar (2× magnification); bronchiolar epithelium, 50 μm scale bar (20× magnification); alveolar septa, 20 μm scale bar (40× magnification).

Following necropsy, we collected tissue sections from the most notably affected right and left lung lobes to examine the presence of histopathological changes in pulmonary tissues on a 17-point scale. In regard to histopathological scores, as expected, the three MV/NC pigs did not show any microscopic lesions in the lungs, besides the occasional presence of minimal to mild bronchus-associated lymphoid tissue hyperplasia (**Figures 5Di–iii**). Similar to gross lesions, the unvaccinated challenged control pigs showed mild microscopic pulmonary pathology (**Figures 5Div–vi**). Vaccination with GA19 displayed optimal performance and induced complete protection from the development of histopathological lesions following homologous challenge (**Figures 5Dvii–ix**).

On the other hand, we observed that all four mismatched vaccination regimens enhanced pulmonary pathology and demonstrated statistically significant increases in the composite histopathological score compared to MV/C animals (*p* < 0.05 for CA09, *p* < 0.001 for MN21 and heterologous prime-boosted group, and *p* < 0.0001 for bivalent vaccine group) (**Figure 5A**). While the histopathology score measured in right lung lobes was significantly higher in all four mismatched vaccine groups compared to MV/C pigs, we noted that microscopic lesions in left lung sections were found significantly enhanced only in the bivalent and heterologous-boosted groups (*p* < 0.001) but not in the MN21 and CA09 vaccine groups (**Figures 5B, C**, respectively). The difference in the left lung score in the MN21 group compared to the MV/C group approached but did not reach statistical significance (*p* = 0.0643).

Regarding histopathological lesions, MN21 and CA09 vaccinated pigs showed mild to moderate bronchial and bronchiolar epithelial attenuation and necrosis, along with moderate alveolar septal mononuclear inflammation (**Figures 5Dx–xii, xii–xv**, respectively). However, histopathological lesions noted in lung sections from the bivalent and heterologous prime-boosted groups were more widespread and pronounced than the mismatched monovalent CA09 and MN21 vaccine groups. Specifically, we observed that bivalent vaccination and heterologous prime-boosting induced severe multifocal to locally extensive necrotizing bronchitis and bronchiolitis that was accompanied by broadly prominent lymphocytic peribronchiolar and perivascular cuffing (**Figures 5Dxvi–xviii, xix–xxi**, respectively). In addition, increased mild to moderate subepithelial mixed lymphocytic and suppurative infiltration, predominantly of peribronchiolar distribution, was present. Alveolar septal thickening was found to be moderately to markedly higher in both groups and we occasionally noted large amounts of alveolar luminal and interlobular seroproteinaceous fluid (edema) and hemorrhage.

Overall, these data suggest that heterologous-boosting and bivalent vaccination enhanced gross and microscopic pulmonary pathology to scores even higher than the monovalent heterologous vaccine groups compared to MV/C control pigs.

## Discussion

Traditionally, influenza vaccination strategies in both human and animal sources have deployed homologous prime-boost vaccination platforms to control virus infections. A limited number of studies have investigated heterologous prime-boost vaccination strategies against influenza infection in swine. To date, heterologous prime-boosting approaches have shown the potential to expand HAI titers against influenza viruses possessing an HA that falls within antigenically distinct phylogenetic clades from the vaccine HA (22–25). However, the vast majority of these studies have focused primarily on the assessment of monovalent experimental H3N2 (22, 24) or multivalent commercial H1–H3 vaccines (23, 25). Only one study to date, a recently published study by Parys et al., has examined the immunogenicity of a heterologous prime-boosting approach by utilizing H1 vaccines containing viruses circulating in European pig populations (49, 50).

Here, we examined the immune responses and protective efficacy induced by an H1-specific heterologous prime-boost vaccination approach based on contemporary North American swine influenza strains. In the current study, while we observed that heterologous prime-boosting increased the breadth of HAI responses against antigenically related influenza variants compared to the monovalent vaccines, this approach failed to expand serological responses against the antigenically distant delta and delta-like H1 influenza strains. However, the most striking finding was that heterologous prime-boost vaccination exacerbated clinical disease and pathology following challenge with the delta2 GA19 virus.

First, we demonstrated that homologous prime-boost monovalent vaccination induced superior HAI titers against viruses that were included in the original vaccine formulation compared to both the bivalent and heterologous prime-boost vaccination (**Figure 1**; **Table 2**). In particular, the mean levels of antibodies measured in bivalent vaccinates post-day 35 pp, while still shown to be considerably above the seroprotective cutoff titer, were significantly lower compared to the corresponding monovalent vaccinated pigs against the homologous CA09 and MN21 vaccine viruses, except for day 35 against the CA09 virus (**Figures 1B, C**). On the other hand, we observed that heterologous prime-boosting evoked antibody titers against the prime A/California/07/2009 virus comparable to monovalent CA09 prime-boosting at all sera collection time points (**Figure 1B**). Interestingly, however, heterologous prime-boost vaccination failed to induce similar levels of antibodies against the boost vaccine virus A/swine/Minnesota/A02636116/2021 as compared to the prime CA09 virus and performed significantly inferiorly compared to both the monovalent MN21 and bivalent vaccine groups (**Figure 1C**).

These results are in line with previous prime-boost studies demonstrating that the sequential order of antigens administered in the regimen influences the immunogenicity and to an extent the protective efficacy of this approach against viruses that are antigenically distinct from the prime or boost vaccine strains (22, 23, 25, 51). This could be attributed to a phenomenon that has been primarily characterized in humans and to a lesser extent in swine called antigenic imprinting or back-boosting (52–56). The concept refers to the observation that secondary heterologous exposures often favor a memory recall and maintenance of enduring immunity preponderantly against influenza variants that are antigenically related to the priming antigen and at a lower level against subsequently encountered antigens (22, 57–59). Indeed, we subsequently observed in our study that heterologous prime-boost vaccination promoted the induction of superior HAI antibody responses against influenza strains that are closely related to the CA09 prime vaccine virus including representative human and swine pandemic HA strains (1A.3.3.2) and gamma clade (1A.3.3.3) swIAVs. Conversely, in sera from heterologous prime-boosted vaccinates, we detected a reduction of up to fourfold and twofold in HAI titers against the alpha clade viruses (1A.1.1) that are homologous to the booster vaccine strain (MN21), as compared to pigs vaccinated with monovalent MN21 WIV and the bivalent vaccine, respectively (**Table 2**).

Previous vaccination studies in swine have observed limited to no HAI cross-reactivity between isolates of different swIAV lineages (46, 60, 61) A similar trend has been observed in the present study; serologic cross-reactivity was in most cases below the seroprotective cutoff value with viruses belonging to antigenically distant lineages to the WIVs included in the original vaccine formulations. However, we noted that sera from pigs vaccinated with the CA09 WIV showed mild cross-reactivity against contemporary gamma and beta clade H1 viruses. Additionally, both the bivalent and the heterologous prime-boost approach in our study failed to expand the scope of immune responses against the antigenically distant delta and delta-like swIAV isolates (**Table 2**). This is in agreement with the recently published European H1 prime-boost study that demonstrated that WIV heterologous prime-boosting did not broaden the cross-reactivity of antibody responses against antigenically distinct swine influenza variants compared to the corresponding monovalent vaccine (49).

While the HAI assay remains the gold standard method for assessing HA-targeting antibody responses following influenza vaccination or infection, a previous study by Leuwerke and colleagues noted that the erythrocyte agglutination ability of viruses tested may affect the sensitivity of the HAI assay (62, 63). In addition to humoral immunity, vaccine-induced cell-mediated responses have been previously shown to be critical in mediating effective virus clearance (64). Further investigation of serological and cell-mediated immune responses through serum neutralization assays, whole virus ELISA, and ELISPOT assays could provide valuable information that may prove pertinent in the interpretation of our results (18).

The main objective of currently developing novel influenza vaccine technologies in human and animal health is the induction of broadly cross-reactive antibodies not only against circulating drifted seasonal or enzootic virus strains but also against emerging influenza variants with pandemic potential (65). In order to overcome the ever-increasing antigenic diversity, numerous novel platforms have focused their attention on the HA stem domain. This region represents a promising antigenic target for broadly protective influenza vaccine development, given that is considered relatively conserved compared to the highly plastic and mutable globular head domain of the HA (66–68). Along the same lines, previous prime-boost studies have presumed that heterologous prime-boosting has the potential of inducing broader cross-reactive immune responses against antigenically distinct IAVs by targeting conserved subdominant epitopes on the HA protein that are shared between the heterologous vaccine strains (22, 69, 70). However, in our study, we observed that the prime-boost approach, rather than enhancing cross-protection, worsened the clinical and pathology outcome after challenge with the antigenically mismatched GA19 virus.

Interestingly, cross-reactive antibodies specific to the conserved epitopes of HA conferred by inactivated vaccines have been previously shown to be implicated in the induction of vaccine-associated enhanced respiratory disease (VAERD) in swine (71, 72). VAERD is most commonly observed in vaccinated pigs following a challenge with a homosubtypic mismatched strain to the vaccine virus, and this phenomenon is characterized by an increased severity in clinical disease and pulmonary pathology (28, 73–75). Previous VAERD studies in swine have traditionally reproduced this phenomenon under experimental conditions by consistently deploying mismatched H1 swIAVs that have an HA of either of 2009 pandemic or delta1-lineage origin as their vaccine and challenge antigens. Additionally, these reports have routinely used monovalent WIV oil-adjuvanted vaccines to incite VAERD (71, 73–75). Notably, however, Parys and colleagues in their recently published study did not observe VAERD in animals immunized with a homologous prime-boost A/California/04/2009/H1N1 vaccination regimen that were later challenged with the mismatched delta1 A/swine/Illinois/A01047020/2010 H1N2 strain. Additionally, they did not observe VAERD in any of the heterologous prime-boosted animals included in their study (49). To our knowledge, the VAERD phenomenon has never been reported before in heterologous prime-boost vaccination studies. This could be attributed to the fact that previous studies that investigated protection conferred by heterologous prime-boosting consistently conducted intranasal inoculation of pigs, which often causes mild respiratory disease and pathology, and fails to reproduce VAERD (22).

In our study, we observed that both the bivalent and heterologous prime-boosted vaccine groups demonstrated increased mean clinical scores following challenge compared to MV/C animals and performed worse over a prolonged period of time than the mismatched monovalent vaccine groups (**Figure 2**). Overall, our results are in agreement with previous findings that demonstrated prolonged clinical disease in VAERD-exhibiting swine (73, 76, 77). To further characterize clinical protection, we evaluated the differential cytology profile in the BALf following challenge between pigs immunized with the different vaccination strategies. We have previously shown that non-vaccinated pigs following uncomplicated influenza infection demonstrate BALf neutrophilic infiltration (30). Interestingly, in the current study, we noted that regardless of the regimen used, mismatched vaccinated pigs demonstrated cytology profiles that were different from those of MV/C animals and were characterized by a primary lymphocytic or mixed lymphocytic–neutrophilic infiltration (**Figure 3**). In contrast, we observed that the vast majority of cells in the BALf of GA19 vaccinates were macrophages, with the presence of only sparse neutrophils and lymphocytes, and the BALf of GA19 vaccinates demonstrated a similar cytology status to non-infected healthy animals. Increased percentages of lymphocytes in the BALf cytology of the mismatched vaccine groups compared to MV/C pigs could be ascribed to an intensified transmigration of these cells from peripheral organs to the inflamed lower respiratory tract, based on the observation that these animals also demonstrated increased mean pulmonary pathology scores (78). Another potential explanation is enhanced activation of primed T cells that recognize conserved epitopes shared with the mismatched vaccine strains (79). Further research into the origin of this increased lymphocytic component is needed to delineate its role in disease enhancement.

Similar to BALf cytology, we observed in lung histology that the majority of pigs from the mismatched vaccine groups showed moderate to marked submucosal lymphocytic infiltration, which was mild or absent in MV/C pigs (**Figure 5**). Overall, these results are suggestive of immunopathological pulmonary lymphocytosis, a phenomenon that has been previously described in humans as a consequence of immune complex-mediated severe 2009 pandemic influenza disease (80). It remains to be determined whether a similar mechanism induces these types of phenomena in our study and if the large numbers of lymphocytes observed in BALf cytology and lung histology mediated a critical role in the exacerbation of lung pathology. In addition to increased accumulation of lymphocytes, we observed intensified alveolar septal thickening and necrotizing bronchitis and bronchiolitis that ranged from moderate to marked in the majority of animals in these groups. Interestingly, while we did not detect any substantial differences in the mean composite and right lung scores between the different mismatched vaccine groups, we observed that only the bivalent and heterologous prime-boosted group demonstrated statistically significant differences in the left lung scores compared to the MV/C animals (**Figure 5**).

Khurana et al. previously suggested that non-neutralizing antibodies that target the conserved stem domain of the HA can increase virus infectivity by enhancing the fusion kinetics of heterologous viruses, and are implicated in the pathogenic mechanism responsible for the induction of VAERD (71). However, enhanced virus shedding and pulmonary virus replication is not a consistent finding in earlier VAERD studies, although it has been reported previously (7, 81, 82). In the current study, we noted that, in addition to the severe pulmonary pathology observed in all mismatched vaccine groups, which is consistent with previous VAERD studies (28, 73, 74, 83, 84), mismatched regimens failed to reduce virus shedding following challenge with the GA19 virus (**Figure 4A**). Moreover, we observed that the virus load in respiratory tissue homogenates of these groups was comparable to that of MV/C pigs (**Figures 4B–D**). Interestingly, we noted that virus titers in respiratory tissues from pigs immunized with the heterologous prime-boost regimen consistently trended higher and, particularly in left lung samples, were found to be significantly increased compared to mock-vaccinated pigs (**Figure 4E**).

In addition to swine, inactivated influenza vaccine-induced immune responses have also been implicated as the cause of enhanced pulmonary disease in humans and other mammals. Several epidemiological investigations have supported the link between receiving the 2008–2009 trivalent inactivated vaccine and an increased risk of medically attended illness following infection with the heterologous pandemic 2009 H1N1 strain (84–86). Uncovering the pathogenic mechanisms that induce VAERD is crucial in the pursuit of advancing broadly protective human influenza vaccines. This is particularly pertinent for ensuring the safety of “universal” vaccine platforms, which consistently focus on conserved epitopes on the HA protein (68).

In conclusion, we showed that heterologous prime-boost vaccination failed to extend the scope of HAI cross-reactivity against antigenically distinct influenza variants. Additionally, while the differences per parameter were not significant, overall heterologous-boosted pigs demonstrated higher clinical disease severity, pulmonary virus load, and lung pathology than the pigs immunized with the mismatched monovalent vaccines. Further research is needed to fill the gaps in existing knowledge and to deepen our understanding of the deregulatory mechanisms that play a part in exacerbating influenza disease following vaccination, particularly mechanisms such as antibody-dependent enhancement of influenza virus entry and cell-mediated and proinflammatory cytokine responses that can set off this phenomenon.

## Data availability statement

The raw data supporting the conclusions of this article will be made available by the authors, without undue reservation.

## Ethics statement

The animal study was approved by Auburn University Institutional Animal Care and Use Committee (IACUC), IACUC Protocol 2022-4095. The study was conducted in accordance with the local legislation and institutional requirements.

## Author contributions

VP: Conceptualization, Data curation, Formal Analysis, Investigation, Methodology, Writing – original draft, Writing – review & editing, Software, Visualization. PN: Investigation, Methodology, Software, Writing – review & editing. MN: Investigation, Methodology, Data curation, Formal Analysis, Writing – review & editing. RN: Data curation, Formal Analysis, Investigation, Methodology, Writing – review & editing. JN: Investigation, Methodology, Writing – review & editing. PS: Investigation, Methodology, Writing – review & editing. SP: Investigation, Methodology, Writing – review & editing. SC: Data curation, Investigation, Writing – original draft. IP: Investigation, Methodology, Validation, Writing – review & editing. ST: Conceptualization, Funding acquisition, Resources, Supervision, Writing – review & editing. CK: Conceptualization, Funding acquisition, Resources, Supervision, Data curation, Formal Analysis, Investigation, Methodology, Project administration, Writing – original draft, Writing – review & editing.
