# Supplementary material for: Heterologous prime-boost H1N1 vaccination exacerbates disease following challenge with a mismatched H1N2 influenza virus in the swine model
Source: Front Immunol. 2023 Oct 20;14:1253626. doi: 10.3389/fimmu.2023.1253626 (PMC10623127; doi:10.3389/fimmu.2023.1253626)
Supplement: Supplementary file 1 [file DataSheet_1.docx]

## Supplemental Material

### Supplemental Table 1.

| **Supplemental Table 1:**  **Amino acid homology of swine and human influenza virus strains used in HAI assays with the HA of the vaccine strains** | | | | |
| --- | --- | --- | --- | --- |
|  | | Vaccine Strains | | |
|  | Designated Clade/ Global Nomenclature | A/California/07/2009 (H1N1) | A/sw/Minnesota/Α02636116/2021 (H1N1) | A/swine/Georgia/A027480/2019 (H1N2) |
| A/swine/Minnesota/Α02636116/2021/H1N1 | alpha/1A.1.1 | 83.58% | 100% | 77.52% |
| A/swine/North Carolina/A02246984/2021/H1N1 | alpha/1A.1.1 | 83.57% | 97.16% | 76.81% |
| A/swine/South Dakota/A01823237/2015/H1N2 | alpha/1A.1.1 | 84.98% | 93.44% | 76.99% |
| A/swine/Kansas/A01785470/2018/H1N1 | beta/1A.2 | 89.22% | 82.16% | 75.80% |
| A/swine/Missouri/A01432837/2013/H1N2 | beta/1A.2 | 89.93% | 83.04% | 76.86% |
| A/swine/Indiana/A01732425/2016/H1N1 | gamma/1A.3.3.3 | 92.05% | 82.16% | 78.62% |
| A/swine/North Carolina/A02245704/2020/H1N1 | gamma/1A.3.3.3 | 89.75% | 81.98% | 76.50% |
| A/swine/South Dakota/A01349306/2013/H1N1 | gamma2/1A.3.2 | 92.23% | 82.51% | 78.45% |
| A/California/07/2009/H1N1 | pdm/1A.3.3.2 | 100% | 83.58% | 77.92% |
| A/swine/Colorado/A02635828/2021/H1N1 | pdm/1A.3.3.2 | 95.23% | 83.04% | 77.39% |
| A/swine/Oklahoma/A01290605/2013/H1N1 | delta1b/1B.2.2.1 | 77.74% | 76.64% | 87.96% |
| A/swine/Iowa/A02431617/2019/H1N1 | delta1a/1B.2.2.2 | 78.27% | 76.45% | 87.61% |
| A/swine/Illinois/A02214842/2017/H1N2 | delta2/1B.2.1 | 78.80% | 76.28% | 95.22% |
| A/swine/North Carolina/A02245213/2019/H1N1 | delta2/1B.2.1 | 77.74% | 76.28% | 94.87% |
| A/swine/Georgia/27480/2019/H1N2 | delta2/1B.2.1 | 77.52% | 77.92% | 100% |
| A/Puerto Rico/8/1934/H1N1 | delta-like/1B.2 | 81.27% | 79.47% | 84.42% |
| A/New Caledonia/20/1999/H1N1 | delta-like/1B.2 | 79.86% | 78.41% | 92.57% |
| A/Brisbane/59/2007/H1N1 | delta-like/1B.2 | 79.33% | 77.70% | 92.04% |
| A/Michigan/45/2015/H1N1 | pdm/1A.3.3.2 | 96.82% | 83.57% | 77.74% |
| A/Michigan/272/2017/H1N1 | pdm/1A.3.3.2 | 96.29% | 83.39% | 77.74% |
| A/Wisconsin/588/2019/H1N1 | pdm/1A.3.3.2 | 95.05% | 83.39% | 77.21% |

### Supplemental Figure 1.


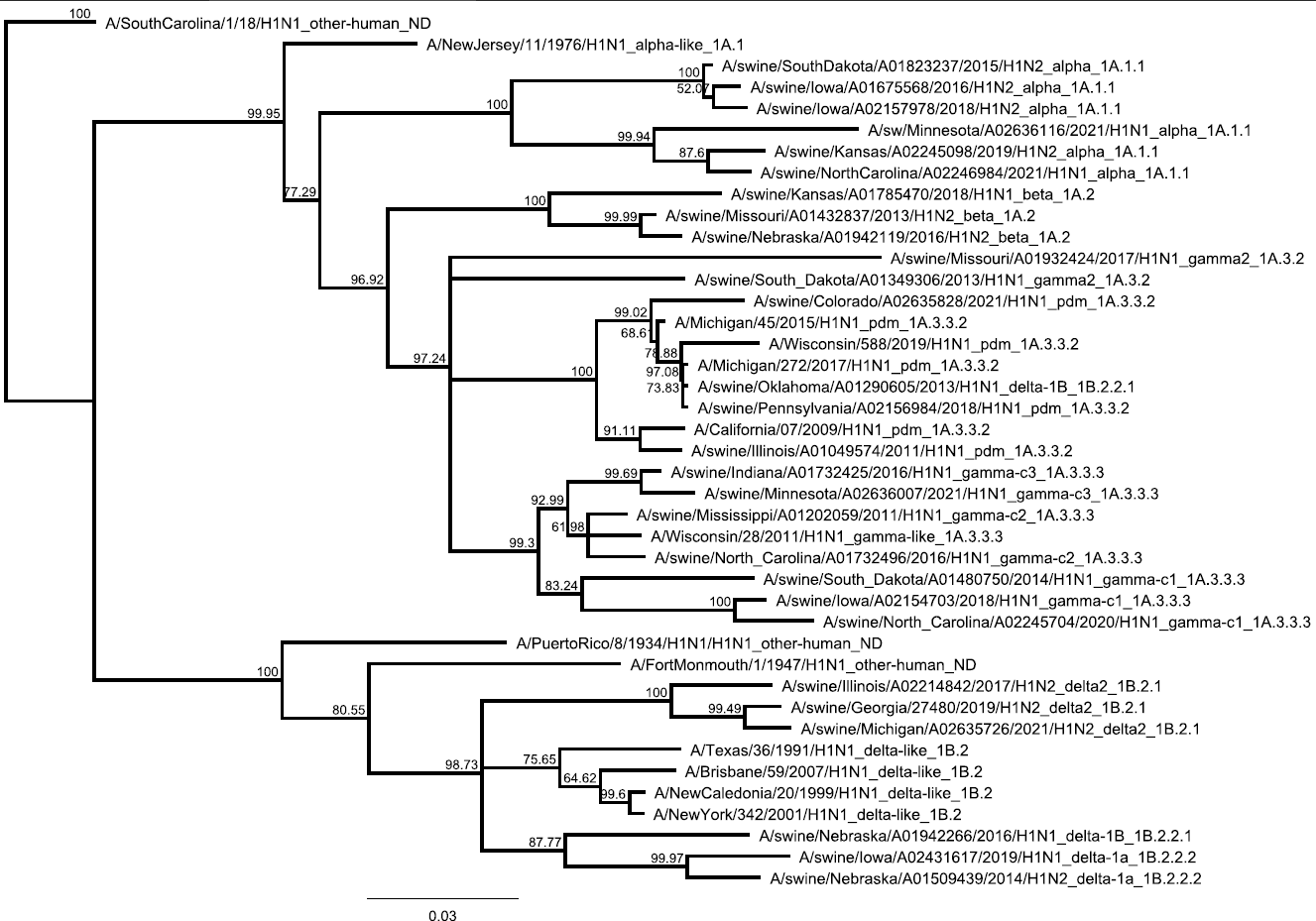


| ***ND*** non-defined, ***pdm*** pandemic |
| --- |

**Supplemental Figure 1.** **Phylogenetic tree showing classification and antigenic relationship of the HA gene segment between the influenza strains employed in this study.**

A phylogenetic tree was constructed based on amino acid sequences of representative H1 North American swine influenza viruses and human influenza viruses isolated over the last century. Amino acid sequences were aligned using MAFFT version 7 and edited using Geneious Software. In Geneious, we created a neighbor-joining tree using Jukes-Cantor genetic distance model and A/South Carolina/1/18/H1N1_other-human_ND as an outgroup. The tree was resampled 1,000,000 times using Bootstrap as a resampling method. All branch lengths are drawn to scale. The scale bar indicates the number of amino acid changes per site. Vaccine viruses are highlighted in blue color. Viruses employed in the HAI assays are labelled with a red mark.
